# Establishment of *Eimeria tenella* (local isolate) in chicken embryos

**DOI:** 10.1051/parasite/2012193285

**Published:** 2012-08-15

**Authors:** L. Jiang, Q. Zhao, S. Zhu, H. Han, H. Dong, B. Huang

**Affiliations:** 1 Key Laboratory for Animal Parasitology, Ministry of Agriculture, Shanghai Veterinary Research Institute, Chinese Academy of Agricultural Sciences No. 518 Ziyue Road Minhang District, Shanghai 200241 China

**Keywords:** *Eimeria tenella*, chicken, embryo, *in vitro* cultivation, vaccine, *Eimeria tenella*, poulet, embryon, culture *in vitro*, vaccin

## Abstract

Development of an *in vitro Eimeria (E.) tenella* model could be valuable as a tool for vaccine, coccidiostats or molecular biology research. 1.0 × 104 sporozoites per 0.1 mL were inoculated into the allantoic cavity of ten-day-old chicken embryos. The complete lifecycle of *E. tenella* was accomplished in eight-nine days at 37 °C and 70% humidity. The addition of 100 U insulin to the embryos could remarkably improve the output of oocysts. The development of the parasite within the embryos was systematically observed, allowing guidelines to be set regarding the appropriate times at which different developmental stages of the parasite may be sampled.

## Abstract (fr)

Le développement d’un modèle *in vitro* d’*Eimeria (E.) tenella* pourrait être précieux pour la production d’un vaccin, de coccidostats ou la recherche en biologie moléculaire. Dans ce but, un inoculum de 1,0 × 104 sporozoites/0,1 mL a été injecté dans la cavité allantoidienne d’embryons de poulet agés de dix jours. Le cycle complet d’*E. tenella* était effectué en huit à neuf jours à 37 °C et 70 % d’humidité. L’addition de 100 U d’insuline pouvait augmenter de façon spectaculaire la production des oocystes chez les embryons. Le suivi sytématique du développement du parasite chez les embryons a permis de définir les périodes de prélèvement de ses différents stades.

Avian coccidiosis, a disease of major economic importance in intensively managed poultry, is caused by the replication within epithelial cells of the intestine of up to seven species of apicomplexan (coccidian) parasites that belong to the genus *Eimeria* (Shirley *et al.*, 2004). The life-cycles of *Eimeria* spp. are completed within a single host and comprise discrete, expansive phases of asexual reproduction followed by a sexual phase that culminates in the production and excretion into the feces of large numbers of oocysts (Fernando, 1990). *E. tenella* is the most prevalent and pathogenic of these species (Han *et al.*, 2010). Cultivation of *E. tenella in vitro* has become increasingly important in recent years as a supplement or a substitute for *in vivo* experiments. Cell and chicken embryo cultivation are the two methods used for the routine reproduction of *E. tenella in vitro*. Initial attempt to cultivate *E. tenella* in embryos was performed by Long (1965) whereby sporozoites of *E. tenella* were reported to adapt successfully in the chorioallantoic membrane (CAM) of chicken embryos. The use of this approach has since proved valuable.

The chicken embryo was used as a test to examine the effects of drug treatment on the viability of sporozoites of *Eimeria* spp. (Long & Jeffers, 1982; Ball *et al.*, 1990). A live attenuated vaccine, which contains an egg-adapted line of *E. tenella* is widely used for the prevention of coccidian infection (Shirley and Bedrnfk, 1997). Furthermore, gametocytes have been obtained from embryos given protection against coccidiosis (Hafeez *et al.*, 2006, 2007; Akhtar et al., 2006). However, few reports have thoroughly investigated methods of embryo cultivation in recent years, especially in China.

The present paper reports the establishment of an embryo cultivation system for *E. tenella* and identifies criteria that proved important in maximizing the yield of oocysts obtained. The complete life-cycle of *E. tenella* in the CAM of chicken embryos is also described.

## Materials and Methods

### Parasites

The Shanghai strain of *E. tenella* was isolated from a sample collected in a chicken farm in Shanghai, China and has been maintained in Shanghai Veterinary Research Institute, Chinese Academy of Agricultural Sciences (Huang *et al.*, 1993). Parasites were propagated as previously described (Tomley *et al.*, 1997). *E. tenella* was amplified by passage through two-week-old coccidian-free chickens and the resulting oocysts were obtained from the cecal contents of chickens eight days post-infection (p.i.). The oocysts were incubated in 2.5 % potassium dichromate solution to induce sporulation. After sporulation, the sporulated oocysts were sterilized by placing in 30 % (v/v) sodium hypochlorite for 30 min and centrifuged at 600 g for ten min. Oocysts were collected from the top of the supernatant and washed several times in sterile PBS. Sporozoites were recovered from cleaned sporulated oocysts by *in vitro* excystation and were purified by chromatography over columns of nylon wool and DE-52 cellulose (Shirley, 1995). They were then incubated in PBS containing 500 units of penicillin and streptomycin per mL at 37 °C for one hour with an appropriate concentration.

### Embryo Injection

Viable day-old chicken embryos were maintained at 37 °C and 70 % humidity in an incubator. Candling was performed to determine the status of the embryo and to mark the position for injection. A 0.1 mL suspension of sporozoites was inoculated into the allantoic cavity and the embryos were placed in an incubator at 37 °C and 70% humidity for eight-nine days. During this period, embryos were observed every day and dead specimens were removed. Deaths within 24 hours of inoculation were deemed to be due to bacterial contamination and these samples did not contribute to the data subsequently analyzed.

### Oocysts Quantitation

The chorioallontoic membrane and allantoic fluid of embryos that survived inoculation were collected into a volume of 2.5 % potassium dichromate solution using a tissue crushing machine to crush and filter the samples through a 100 mesh copper screen. 4 mL filtrate was centrifuged at 1,300 g for ten min, the supernatant was discarded and 4 mL of saturated brine was added. The number of oocysts was counted three times in each sample and expressed using the McMaster’s method with the equation:

$$\mathrm{Quantity}\;\mathrm{of}\;\mathrm{oocysts}\;\mathrm{per}\;\mathrm{embryo}=\frac{\mathrm{McMaster’}s\;\mathrm{oocyst}\;\mathrm{number}\times \;\mathrm{volume}\;\mathrm{of}\;\mathrm{collected}\;\mathrm{solution}}{0.15\;\times \;\mathrm{number}\;\mathrm{of}\;\mathrm{embryos}}$$

### Obtaining Polyclonal Sera Raised Against Sporozoites

The antibody used for immunofluorescence analysis was produced by our laboratory.The proteins extracted from sporozoites were used to immunize two-month-old female rabbits by intraperitoneal (i.p.) injection with 200 μg of proteins emulsified in Freund complete adjuvant (Sigma). Rabbits were boosted three times at intervals of two weeks with proteins emulsified in Freund incomplete adjuvant. Eight days after the final immunization, serum was separated from the rabbits’ blood.

### Selection of Optimum Embryo Age for Inoculation

The age of embryos may influence the ability of sporozoites to invade the CAM and develop. To examine this, eight-eleven-day-old embryos were injected with sporozoites in order to select the best inoculation age of embryos. Following sporogony, 100 oocysts were counted under a microscope to distinguish sporulated and unsporulated oocysts for showing the quantitation of oocysts from embryos. The experiment was repeated for three times, and results from all three experiments were analyzed.

### Selection of Optimum Dose for Sporozoite Inoculation

Eggs provide a microenvironment for sporozoites to complete their life-cycle. Inoculation of an optimum dose of sporozoites should lead to a balance between host and parasite that allows the development and output of oocysts to be maximized. To identify the best inoculation dose, 0.1 × 104 – 50 × 104 sporozoites were injected into ten-day-old embryos respectively, and the experiment was repeated for three times.

### Insulin Addition

The hormone insulin is central to the regulation of glucose metabolism in the body and helps cells to take glucose from the blood. To assess whether insulin can improve the output of oocysts, concentrations of 1,000, 100 or 10 U insulin (Sigma) were added to the suspension, which contained 1 × 104 sporozoites per mL and then administered to ten-day-old embryos. The experiment was repeated for three times.

### Development of the Parasite

After the injection of sporozoites into the allantoic cavity, the parasites were detected only in the chorioallantoic membranes, and not in the embryo itself. To examine the development of the parasites in the embryos, 50 embryos were infected with sporozoites and the CAM of the infected embryos was obtained at different time points over a period of nine days and spread on glass slides, air-dried, fixed in 2% paraformaldehyde and stained with immunofluorescence, the periodic acid sehiff (PAS) approach (Hofmann, 1990), while some slides were observed directly by microscope.

## Results

### Selection of Optimum Embryo Age for Inoculation

Ten-day-old embryos showed a lower mortality and higher rate of oocyst production than embryos of other ages. Furthermore, as the development of sporogony progressed, ten-day-old embryos were higher than those of eleven-day-old, and no significant difference from those of eight and nine-day-old ones. We choose ten-day-old embryos for subsequent inoculations (Table I).

**Table I.** The affect of embryo age for inoculation.

Embryos age (day)	Injected embryos	Survived embryos	Mortality rate (%)	Oocysts production (× 10^4^)	Sporogeny rate (%)
8	30	15	50.0	1.3	80.2
9	30	17	43.3	1.0	80.4
10	30	27	10.0	2.1	78.2
11	30	24	20.0	0.5	40.6

### Selection of Optimum Dose for Sporozoite Inoculation

As shown in Table II, inoculating 1 × 10^4^ sporozoites per embryo resulted in a significantly lower mortality rate and higher rate of oocyst production than other doses.

**Table II.** Influence of the number of sporozoites inoculated per embryo.

Dose of sporozoites (× 10^4^)	Injected embryos	Survived embryos	Mortality rate (%)	Oocysts production (× 10^4^)
0.5	30	28	6.7	2.16
1.0	30	28	6.7	2.81
5.0	30	23	23.3	0.78
10.0	30	20	33.3	0.16
20.0	30	16	46.7	0.11
50.0	30	8	73.3	1.00

### Insulin Addition

**Fig. 1.**
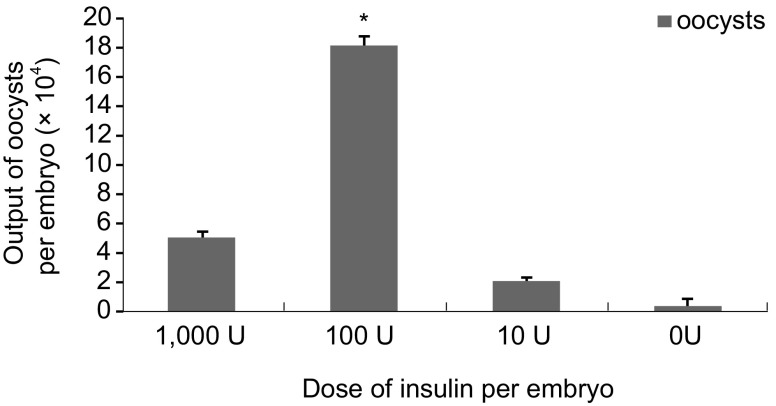
Yeilds of *E. tenella* in embryos with different concentrations of insulin. * p < 0.05, n = 3.

The addition of 100 U insulin improved the output of oocysts (1.8 × 10^5^ per embryo) compared to the other concentrations. At this level, embryos developed well and no mortality occurred, except those were contaminated with bacterium (Fig. 1).

### Development of Parasites

**Fig. 2.**
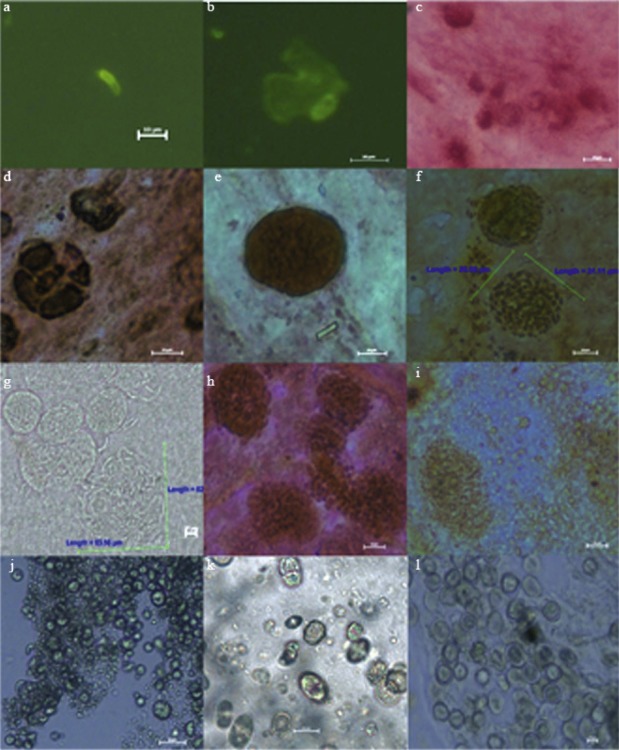
Sporozoites, trophozoites, schizonts, gamonts and oocysts in embryos at various times after inoculation (a-l). (a) 2 h p.i., sporozoites; (b) 12 h p.i., sporozoites in the CAM cell; (c) 24 h p.i., trophozoites; (d) 48-h p.i., immature schizonts; (e) 60 h p.i., mature first-generation schizonts; (f) 72 h p.i., mature first-generation schizonts and merozoites; (g) 96 h p.i., second-generation schizonts and merozoites; (h) 120 h p.i., second-generation schizonts and merozoites; (i) 144 h p.i., immature macrogametes and microgametocytes; (j) 168 h p.i., mature macrogametes and microgametocytes; (k) 192 h p.i., oocysts and immature oocysts; (l) nine-days p.i., mature unsporulated oocysts.

The complete life-cycle of *E. tenella* was observed (Fig. 2 a–l). Sporozoites could be seen on the CAM at 2 h p.i. (a), after which they invaded the CAM cells themselves and adopted a round shape at 12 h p.i. (b). At 24 h p.i., these intracellular sporozoites were seen to be surrounded by a parasitophorous vacuole and developed into trophozoites (c). Immature schizonts were observed at 48 h p.i. (d). At 60 h p.i., mature first-generation schizonts had increased both in size and in the number of nuclei present (e). At 72 h p.i., mature schizonts were accompanied by numerous PAS-positive merozoites, many of which were released at this stage (f). At 96 h p.i., large numbers of second-generation schizonts measuring 16-46 × 18- 52 μm were observed (g), which contained hundreds of merozites arranged in a radial form. Released merozoites moved rapidly amongst the schizonts and measured around 16 × 2 μm in size. The release of merozoites continued at 120 h p.i. (h) and began to develop into macrogametes and microgametocytes by 144 h p.i. (i). At 168 h, both mature macrogametes and microgametes were evident. These were round in shape and measured about 8 × 7 μm and 2 × 3 μm, respectively (j). Immature oocysts measuring 21 × 17 μm were present in clusters at 192 h p.i. (k). Mature unsporulated oocysts could be obtained from the CAM at 9 days p.i. (l).

## Discussion

*Eimeria* parasites of the fowl usually multiply in the host’s intestinal tract and oocysts can be collected from the gut contents or feces. Collection and subsequent purification by physical or chemical methods are regarded as labour intensive tasks. In recent years, embryo cultivation of *Eimeria* spp. has become increasingly important as a supplement to other approaches. Embryos provide an uncontaminated environment for *Eimeria* spp. to complete their life-cycle and can provide endogenous stage materials such as merozoites or gamonts in large numbers. Thus, embryo cultivation can assist in addressing questions in the biological, biochemical and immunological fields of research by providing an *in vitro* system for study.

Several studies have focused on the growth of *E. tenella* on the CAM of chicken embryos, but few have quantified the output of oocysts from the embryos. To optimize the system of embryo culture, quantification of oocyst output is required. The sporozoites of *E. tenella* can be adapted to complete their life-cycle on the CAM of chicken embryos, resulting in the release of free oocysts into the allantoic fluid and in the necrotic material sloughed off the chorioallantoic membranes (Long, 1965). In the present study, these materials were collected from embryos into a specific volume of 2.5 % potassium dichromate solution, which decreased the viscosity of the CAM by weak oxidation, allowing the releasing of oocysts that were counted by McMaster’s method.

The inoculation of sporozoites (1.0 × 10^4^) into 10- day-old chicken embryos resulted in a mortality rate of only around 10%, much lower than the reported rate of 86.4 % (Hafeez, Akhtar and Ayaz, 2006, 2006, 2007). However, mortality was higher in younger embryos or those given a higher dose of sporozoites. In these situations, we thought that embryonic death may be due to other causes rather than parasitemia *per se*. The oocysts used to prepare the sporozoites might be placed in 30% (v/v) sodium hypochlorite for 30 min. This ought to have completely sterilized the samples from any toxic substances originating in the intestine or feces, such as bacteria or “little black” polypides that proliferate rapidly in oocyst suspensions. The incubation temperature of 37 °C was used for parasite’s development in this study, not the normal recommended temperature (39~41 °C) (Long, 1970; Hafeez, 2006). As we know, this temperature was suitable for the development of embryos and, maybe also reduced the mortality of embryos. In the present study, increasing the number of inoculated sporozoites resulted in a reduction in the output of oocysts. This pattern perhaps resulted from the damage to the CAM by high numbers of sporozoites, which caused insufficient cells or nutrition for the parasites to complete their life-cycle.

The hormone insulin is critically involved in the regulation of glucose metabolism and helps cells to take glucose from the blood. As the administration of 0.05 μg/mL insulin promotes cell multiplication and the development of coccidian in chicken kidney cells (Xie *et al.*, 1996), we examined the effect of adding various doses of insulin to sporozoite suspensions inoculated into embryos. A 100 U insulin dosage was associated with an increase in CAM growth and thickness and an increase in oocyst output. In contrast, these effects were not seen with a high dose of 1,000 U, potentially as the CAM may have grown so well that it inhibited sporozoite proliferation.

The development of sporozoites in embryos was systematically observed. The life-cycle appeared to be delayed with a large number of oocysts only appearing after day 9 of infection and the schizogony phase being extended to 48-144 h, which was also shown in Long’s study (1970). The observed schizonts were morphologically similar to second-generation schizonts of *E. tenella* in the coeca of chickens that received sporulated oocysts. In our study, the development of sporozoites did not follow the same pattern as in the earlier work. As a large number of embryos were observed, the pattern of development under these conditions is likely to have been accurately described. The immunofluorescence approach using an antibody specific to sporozoite proteins was valuable in allowing the detection of sporozoites which were otherwise too small to be differentiated from the CAM, whilst not recognizing schizonts and merozoites well which were, in contrast, easily distinguishable.
